# Anxiety Mediates the Relationship between Perfectionism and Insomnia Symptoms: A Longitudinal Study

**DOI:** 10.1371/journal.pone.0138865

**Published:** 2015-10-14

**Authors:** Umair Akram, Jason G. Ellis, Nicola L. Barclay

**Affiliations:** Faculty of Health and Life Sciences, Northumbria University, Newcastle-upon-Tyne, United Kingdom; United Graduate School of Child Development, JAPAN

## Abstract

**Objectives:**

Individuals with insomnia often report aspects of perfectionism and symptoms of anxiety and depression. Investigation of these factors together has been limited. As such, the aim of the present study was to examine the extent to which the association between perfectionism and insomnia symptoms was mediated by anxiety and depression, concurrently and longitudinally.

**Methods:**

Seventy-six members from the general-population participated at baseline. Data from 57 participants were subsequently analysed at twelve-month follow-up. Insomnia symptoms were assessed using The Insomnia Severity Index (ISI). Perfectionism was assessed using two Multidimensional Perfectionism Scales (F-MPS; HF-MPS). Symptoms of anxiety and depression were assessed using The Hospital Anxiety and Depression Scale (HADS). Correlational analysis examined longitudinal associations between perfectionism and insomnia symptoms. Hierarchical regression analysis examined whether significant associations remained after controlling for anxiety and depression.

**Results:**

Baseline insomnia symptoms were associated with future doubts about action. Further, this relationship was mediated by preceding symptoms of anxiety and concurrent symptoms of insomnia. Similarly, baseline insomnia symptoms were also associated with future parental criticism. However this relationship was partially mediated by preceding symptoms of anxiety, and was not mediated by concurrent insomnia symptoms.

**Conclusions:**

Symptoms of insomnia appear to be related to an increase in negative perfectionistic thinking in the form of doubts about action and parental criticism, however these relationships appear to be mediated by symptoms of anxiety. Therefore, treatments for insomnia should address anxiety symptoms with the prospect of preventing the accentuation of aspects of perfectionism due to poor sleep.

## Introduction

Sleep disturbances such as insomnia are influenced by a number of predisposing, precipitating and perpetuating factors, which can be behavioral, biological, environmental, or psychological in nature [1]. Insomnia is arguably the most prevalent sleep disorder, affecting between 9–15% of the general population, and is commonly comorbid with many psychiatric disorders including generalized anxiety, depression, bipolar, obsessive-compulsive and posttraumatic stress disorder [2–6]. Depression and anxiety appear to be the most common psychiatric disorders associated with insomnia [7], and evidence points towards symptoms of anxiety being a greater risk factor for the onset of insomnia [8–10], whereas depression may be a consequence of insomnia [3, 11]. Furthermore, one’s personality may inherently act as an influencing factor in relation to the onset of many psychiatric disorders, and indeed insomnia. Literature concerning personality concurs that individuals with insomnia exhibit increased neuroticism, internalization, anxious concerns, and specific components of perfectionism including doubts about action, parental criticism, concern over mistakes, personal standards, and socially prescribed perfectionism [12, 13–15].

It has been postulated that individuals with perfectionistic traits may exhibit a propensity to be overly concerned with the negative effects of a poor night’s sleep [16]. Such concerns could inadvertently perpetuate the development of a vicious thought cycle consisting of worry, frustration, and negative expectations concerning sleep [17]. As such, perfectionism can be considered to be both a predisposing and perpetuating factor in insomnia.

Perfectionism has been defined as the tendency to set excessively high standards for oneself and to engage in overly critical self-evaluations [17]. Two multidimensional conceptualizations of perfectionism have been proposed and generally accepted, each differing in their approach to distinguishing the constructs of perfectionism [17, 18]. According to Frost and colleagues [17] six dimensions characterize the construct of perfectionism: the propensity to be concerned over and react negatively to mistakes (concern over mistakes); the propensity to doubt ones own performance and actions (doubts about action); the perception that ones parents have high expectations of them (parental expectations); the perception that ones parents are overly critical towards them (parental criticism); the propensity to maintain a high standard of order and organisation (organisation); and the propensity to set and maintain high personal standards (personal standards). Expanding beyond the notion of an overly critical self-view, Hewitt and colleagues [18] conceptualized perfectionism into three dimensions: unrealistic standards for the self (self oriented perfectionism, SOP); unrealistic standards expected of others (other oriented perfectionism, OOP); and the belief that others hold high standards for oneself (socially prescribed perfectionism, SPP). Although these two conceptualizations differ in their approach to perfectionism, considerable similarities between their constructs can be noted. Indeed, a propensity to maintain a high level of personal standards and organization, paired with a tendency to ruminate over doubts and concerns with one’s behavior, appears to be somewhat characteristic of self oriented perfectionism, which encompasses unrealistic self imposed standards and enhanced self monitoring. Moreover, whilst Frost and colleagues [17] highlight the contribution of perceived parental evaluation to perfectionism (i.e. parental expectations and criticism), Hewitt and colleagues [18] propose this approach to be rather restricted, suggesting that the perceived evaluation of other people (i.e. peers, colleagues) should also be taken into account.

Equivocal conclusions have been reported concerning which aspects of perfectionism are specifically associated with sleep disturbances and insomnia. The lack of consensus amongst studies is likely due to varying methodologies, scale choices, and samples (e.g. normal vs. clinical). For example, Lundh and colleagues [16] demonstrated that amongst the Swedish general population, concern over mistakes and doubts about action were positively related to levels of sleep disturbance. Additionally, the authors demonstrated that insomnia patients from a sleep disorders clinic reported greater concern over mistakes, doubts about action, and personal standards in comparison to those from the general population. These findings have partially been replicated by Vincent and colleagues [16] who found that individuals with insomnia report increased concern over mistakes, doubts about action and parental criticism. Moreover, using Hewitt & Flett’s conceptualization of perfectionism, Azevedo and colleagues [14, 15] established that socially prescribed perfectionism was associated with sleep disturbance amongst a sample of undergraduate students. The authors also found socially prescribed perfectionism to be a reliable predictor of sleep disturbance over time.

Despite the inconsistencies between studies, there appears to be evidence of relationships between poor sleep and the multidimensional perfectionism subscales doubts about action, parental criticism, concern over mistakes and personal standards [13], and the multidimensional perfectionism dimension of socially prescribed perfectionism [14, 15]. Interestingly, studies have also demonstrated that concern over mistakes, doubts about action and socially prescribed perfectionism are associated with depressive symptoms [17, 19–24]. Additionally, in comparison to nonclinical controls, patients with unipolar depression have reported greater symptoms of concern over mistakes and socially prescribed perfectionism [25]. Similar results have been noted between anxiety and perfectionism. For example, research using student samples has demonstrated that increased concern over mistakes and doubts about action were associated with higher levels of anxiety symptoms [26, 27]. Further, whereas symptoms of depression often emerge as the consequence of insomnia [3, 11], it appears that insomnia is often preceded by symptoms of anxiety [8–10]. With this in mind, anxiety may act as a precursor to a sleep disturbance due to a progressive increase in worry and ruminative thinking during the pre-sleep period, perhaps with a specific focus on doubts regarding past and future behaviour(s). Consequently, this pattern of thinking could inadvertently contribute to the development of negatively toned cognitive activity, arousal and distress that subsequently leads to a delayed onset of sleep [28]. Moreover, it is likely that the daytime consequences of insomnia impair the ability to adequately cope with social and interpersonal difficulties, as well as the stressors of daily life that could, in turn, precipitate depressive symptoms [29].

Given the inter-relationships between insomnia, perfectionism, anxiety and depression, it is perhaps no surprise that emerging evidence suggests that the association between insomnia and perfectionism may be mediated by anxiety and depression. For example, Jansson-Frӧjmark & Linton 30] note that concern over mistakes appears to be significantly related to pre-existing and future insomnia. However, when emotional distress in the form of anxiety and/or depression was accounted for, these relationships diminished. With this in mind, it appears that understanding the potential mediational role of anxiety and/or depression in the insomnia-perfectionism relationship is important and may have implications for treatments for insomnia. The findings by Jansson-Frӧjmark and colleagues [30] were seminal in establishing a meditational role of anxiety and depression on the relationship between perfectionism and insomnia. That said, as the authors utilized only two perfectionism subscales (concern over mistakes, doubts about action) to assess this relationship, it may be pertinent to examine the full range of perfectionism subscales and dimensions when examining the relationship between perfectionism, anxiety and depression, and poor sleep.

In sum, to the best of our knowledge only two studies have investigated the relationship between insomnia and perfectionism from a longitudinal perspective, demonstrating that increased concern over mistakes and socially prescribed perfectionism appear to be related to future sleep disturbance [15, 30]. Furthermore, only one of those studies has examined the possibility that the relationship between various aspects of perfectionism and insomnia may be mediated by anxiety and/or depression [30]. Given the evidence for inter-relationships between Hewitt and Flett’s conceptualization of perfectionism with insomnia, anxiety and depression, it also appears a worthy line of enquiry to examine the possible meditational role of these traits.

The present study utilized the full range of subscales and dimensions from both conceptualizations of perfectionism to examine the potential bi-directional relationships between insomnia, perfectionism, anxiety and depression amongst a sample of the general population over the course of one-year. Specifically, to determine: i) the extent to which the association between perfectionism and insomnia symptoms were mediated by anxiety and depression, concurrently at both time points; ii) whether perfectionism at baseline was related to future insomnia symptoms after accounting for anxiety and depression; and finally iii) whether insomnia symptoms at baseline was related to an increase in the reporting of perfectionism at follow-up after accounting for anxiety and depression.

## Methods

### Ethics Statement

The study was approved by the Northumbria University Faculty of Health and Life Sciences Ethics Committee, and all participants provided informed consent prior to participation.

### Participants

Participants from the general population were recruited for the study by the use of posters around Northumbria University, emails to students, and social media. Participants who indicated that they conducted shift work, suffered from a disorder of the central nervous system, or had a prior head injury or reported symptoms of a sleep disorder other than insomnia were excluded from the study through the use of an online screening questionnaire. At baseline, 76 participants were included in analyses [mean age = 25.30 years, standard deviation (SD) = 10.36 years, range 19–64; 80% female]. Data from 57 participants were subsequently analysed at follow-up one year later [mean age = 26.11 years, SD = 9.29 years, range 20–60; 84% female]. Participant characteristics concerning ethnicity, work status, and education are reported in Table 1. There were no significant differences in age, sex, socioeconomic status, insomnia symptoms, anxiety, depression or perfectionism between those who responded at follow-up and those who did not (all *p*’*s* > .05).

**Table 1:** Participant characteristics concerning ethnicity, work status and education level.

	Baseline (n = 76)		Follow-up (n = 57)	
	N	%	N	%
Ethnicity				
White	70	92.1	53	93
Asian	6	7.9	4	7
Work Status				
Paid Employment	14	18.4	11	19.3
In Education	61	80.2	45	78.9
Retired	0	0	0	0
Unemployed	1	1.4	1	1.8
Education				
No Education	1	1.3	1	1.8
GCSE	2	2.6	1	1.8
A Level	50	65.8	38	64.9
HND	2	2.6	1	1.8
Degree	7	9.3	6	10.5
Postgraduate Degree	14	18.4	11	19.2

Notes

N, Number of Participants; %, Percentage of Participants; Education, Highest Qualification Attained; GCSE, General Certificate of Secondary Education; HND, Higher National Diploma.

### Measures

#### Insomnia Severity Index

Insomnia symptoms were assessed using The Insomnia Severity Index [31]. The ISI consists of 7 items examining the severity of insomnia symptoms over the past two weeks including difficulty initiating and maintaining sleep, and awakening too early. Items are scored on a 5-point likert scale, with total scores ranging from 0–28. Higher scores represent greater insomnia severity. Assessment of internal consistency yielded a Chronbach’s alpha of .89 at baseline, and .91 at follow-up.

#### Multidimensional Perfectionism Scales

Two Multidimensional Perfectionism Scales were administered in order to assess different aspects of perfectionism (F-MPS [17]; HF-MPS [18]). The F-MPS consists of 35 items assessing six components on 5-point likert scales. The maximum scores of each component are as follows: concern over mistakes (CM) 45; doubts about action (D) 20; parental expectations (PE) 25; parental criticism (PC) 20; organisation (ORG) 30; and personal standards (PS) 35. Higher scores represent a greater tendency towards perfectionism. Assessment of internal consistency yielded a Chronbach’s alpha of .92 for the subscale CM; .80 for D; .86 for PE; .85 for PC; .92 for ORG; and .87 for PS at baseline, and 91 for the subscale CM; .79 for D; .89 for PE; .82 for PC; .92 for ORG; and .86 for PS at follow-up.

The HF-MPS consists of 45 items assessing three dimensions on 7-point likert scales. The three dimensions, self oriented perfectionism (SOP); other oriented perfectionism (OOP); and socially prescribed perfectionism (SPP), each comprise a maximum total score of 105. Higher scores represent a greater tendency towards perfectionism. Assessment of internal consistency yielded a Chronbach’s alpha of .91 for the subscale of SOP; .71 for OOP; and .90 for SPP at baseline, and .93 for the subscale of SOP; .80 for OOP; and .89 for SPP at follow-up.

#### The Hospital Anxiety & Depression Scale

Anxiety and depression were assessed using The Hospital Anxiety and Depression Scale (HADS [32]). The scale consists of 14 fixed choice items (seven for both anxiety and depression) each scored between 0 and 3, and each with a maximum total score of 21. Higher scores on each subscale represent greater anxiety and depression. Assessment of internal consistency yielded a Chronbach’s alpha of .85 for the anxiety subscale, and .73 for the depression subscale at baseline, and .86 for the anxiety subscale, and .78 for the depression subscale at follow-up.

#### Procedure

At baseline, participants completed an online survey assessing insomnia symptoms, perfectionism, anxiety and depression. At follow-up, approximately one year later, participants who had previously participated were contacted via email and were asked to repeat the online survey. Data was collected during December of 2011 and January of 2012 (baseline, T1), and December of 2012 and January of 2013 (follow-up, T2).

#### Statistical Analyses

Normality of the data was initially assessed by histograms. In addition, a series of Shapiro-Wilks tests were also conducted for each variable at both time points in order to confirm a normal distribution (all *p*’s were > .05). Pearson’s bivariate correlations examined concurrent and longitudinal associations between insomnia symptoms, aspects of perfectionism, anxiety, and depression at baseline and follow-up. This was followed by hierarchical linear regression analyses, with insomnia symptoms as the dependent variable, in order to determine whether any significant associations between perfectionism and symptoms of insomnia were mediated by anxiety and depression after controlling for age and sex, concurrently at baseline. Of note, concurrent regression analyses to assess the possibility of mediation were not performed at follow-up as the correlational analyses demonstrated no significant associations between insomnia symptoms and aspects of perfectionism.

Further, longitudinal hierarchical linear regression analyses were performed in order to determine whether established significant associations between insomnia symptoms at baseline and perfectionism at follow-up after were mediated by baseline anxiety, depression, and future insomnia symptoms after controlling for age and sex. For example, age (step 1), sex (step 2), follow-up insomnia symptoms (step 3), baseline insomnia symptoms (step 4), baseline anxiety (step 5) and baseline depression (step 6) were entered as predictor variables, with the perfectionism subscale doubts about action as the dependent variable. Of note, longitudinal regression analyses to assess the possibility of mediation were not performed with insomnia symptoms at follow-up as the dependent variable as the correlational analyses deemed these associations not to be significant. For all analyses, significance was considered at the *p* < 0.05 level (two-tailed).

## Results

Mean scores on insomnia symptoms, perfectionism, and anxiety and depression scores at baseline (T1) and follow-up (T2) are presented in Table 2.

**Table 2:** Mean scores and standard deviations (SD) for insomnia symptoms, anxiety, depression and perfectionism.

	Baseline (n = 76)			Follow-up (n = 57)		
	M	*SD*	Range	M	*SD*	Range
Insomnia Severity Index	6.97	5.08	0–20	7.35	5.62	0–27
Anxiety	7.80	4.41	0–19	7.61	4.52	1–19
Depression	3.45	2.88	0–13	3.77	3.10	0–13
Concern over mistakes	22.47	7.89	9–41	22.02	7.44	9–39
Doubts about action	12.72	3.35	6–20	12.33	3.74	4–20
Parental expectations	12.74	4.68	5–23	12.53	4.64	5–22
Parental criticism	8.63	3.82	4–18	8.23	3.43	4–19
Organisation	22.84	4.72	8–30	23.42	4.53	13–30
Personal standards	22.32	5.93	7–35	21.93	5.97	9–33
Self oriented perfectionism	68.30	16.52	28–102	67.84	17.36	27–104
Other oriented perfectionism	54.42	11.45	25–91	53.47	11.41	28–82
Socially prescribed perfectionism	50.63	14.87	21–87	52.30	15.00	22–87

Notes

Range: Min–Max; Insomnia Severity Index: Possible Range 0–28; Perfectionism: Concern Over Mistakes, Possible Range 9–45; Doubts About Action, 4–20; Parental Expectations, 5–25; Parental Criticism, 4–20; Organisation, 6–30; Personal Standards, 7–35; Self Oriented Perfectionism, 15–105; Other Oriented Perfectionism, 15–105; Socially Prescribed Perfectionism, 15–105; Hospital Anxiety & Depression Scale: Anxiety, Possible Range 0–21; Depression; 0–21.

### Concurrent Results

Concurrent correlational analysis indicated that, at baseline, insomnia symptoms were significantly associated with anxiety (r = .51, *p* = .01), depression (r = .55, *p* = .01), and the perfectionism subscales of doubts about action (r = .26, *p* = .02) and parental criticism (r = .29, *p* = .01). No other perfectionism subscales were significantly associated with insomnia symptoms. In addition, the perfectionism subscales of doubts about action and parental criticism were also associated with anxiety (D: r = .39; PC: r = .34, *p* = .01 respectively) and depression (D: r = .28; r = .34, *p* = .01 respectively). At follow-up, none of the perfectionism subscales were significantly associated with symptoms of insomnia (see Table 3 for all correlations between insomnia symptoms, perfectionism, anxiety, and depression at baseline and follow-up).

**Table 3:** Concurrent correlations between insomnia symptoms, anxiety, depression and perfectionism at baseline and follow-up.

	1	2	3	4	5	6	7	8	9	10	11	12
1. ISI	−	.55**	.49**	.17	.22	.04	.02	.03	.03	-.04	-.18	.12
2. Anxiety	.51**	−	.61**	.41**	.52**	.21	.36**	.17	.20	.32*	-.02	.42**
3. Depression	.55**	.62**	−	.33**	.44**	-.02	.25	.04	.15	.22	.03	.19
4. CM	.14	.35**	.30**	−	.60**	.61**	.62**	.21	.69**	.64**	.40**	.82**
5. D	.26*	.39**	.28*	.60**	−	.38**	.55**	.32*	.45**	.47**	.17	.50**
6. PE	.14	.19	.13	.63**	.40**	−	.50**	.19	.65**	.51**	.32*	.73**
7. PC	.29*	.34**	.35**	.67**	.49**	.59**	−	.07	.37**	.24	.17	.58**
8. ORG	-.01	.11	.14	.26*	.37**	.18	.04	−	.38**	.44**	.44**	.27*
9. PS	-.04	.23*	.18	.75**	.55**	.50**	.43**	.43**	−	.80**	.53**	.69**
10. SOP	-.10	.23*	.13	.65**	.55**	.40**	.35**	.46**	.72**	−	.48**	.64**
11. OOP	-.21	-.02	-.06	.43**	.31**	.39**	.16	.19	.44**	.50**	−	.37**
12. SPP	.08	.30**	.14	.78**	.50**	.75**	.59**	.11	.58**	.59**	.46**	−

Notes

Baseline (n = 76) correlations are displayed below the diagonal; follow-up (n = 57) correlations are displayed above the diagonal.

ISI, Insomnia Severity Index; CM, Concern Over Mistakes; D, Doubts About Action; PE, Parental Expectations; PC, Parental Criticism; ORG, Organization; PS, Personal Standards, SOP, Self Oriented Perfectionism; OOP, Other Oriented Perfectionism; SPP, Socially Prescribed Perfectionism.

*Significant for p-level < .05.

** Significant for p-level < .01.

Linear regression analyses indicated that, at baseline, the perfectionism dimensions of doubts about action and parental criticism significantly predicted baseline insomnia symptoms. However, when anxiety and depression were accounted for, only anxiety and depression were significantly related to insomnia symptoms, with none of the previous perfectionism associations remaining significant (see Tables 4 and 5).

**Table 4:** Concurrent hierarchical linear regression analyses with insomnia symptoms as the dependent variable, and age, sex, doubts about action, anxiety and depression as predictors at baseline.

*Predictors in the model*	R^2^	β	*t*	Sig.
Step 1				
Age	.01	.08	0.72	.47
Step 2				
Age	.01	.08	0.73	.47
Sex		.06	0.55	.59
Step 3				
Age	.09	.14	1.19	.24
Sex		.03	0.28	.78
Doubts About Action		.28	2.44	.02*
Step 4	.28			
Age		.07	0.70	.49
Sex		-.04	-0.38	.71
Doubts About Action		.09	0.81	.42
Anxiety		.48	4.30	.01**
Step 5	.35			
Age		.04	0.35	.73
Sex		.03	0.27	.79
Doubts About Action		.06	0.56	.58
Anxiety		.26	1.92	.05*
Depression		.37	2.90	.01**

Notes

*Significant for p-level < .05.

** Significant for p-level < .01.

**Table 5:** Concurrent hierarchical linear regression analyses with insomnia symptoms as the dependent variable, and age, sex, parental criticism, anxiety and depression as predictors at baseline.

*Predictors in the model*	R^2^	β	*t*	Sig.
Step 1				
Age	.01	.08	0.72	.47
Step 2				
Age	.01	.08	0.73	.47
Sex		.06	0.55	.59
Step 3				
Age	.10	.07	0.63	.53
Sex		.11	0.94	.35
Parental Criticism		.30	2.68	.01**
Step 2				
Age	.28	.05	0.51	.61
Sex		-.01	-0.06	.95
Parental Criticism		.13	1.16	.25
Anxiety		.47	4.23	.01**
Step 3				
Age	.36	.02	0.22	.83
Sex		.05	0.45	.67
Parental Criticism		.08	0.79	.43
Anxiety		.25	1.95	.05*
Depression		.36	2.83	.01**

Notes

*Significant for p-level < .05.

** Significant for p-level < .01.

### Longitudinal Associations

Longitudinal associations indicated that baseline insomnia symptoms were significantly associated with the perfectionism subscales of doubts about action (r = .30, *p* = .02) and parental criticism (r = .32, *p* = .02) at follow-up. No significant associations were found between any of the perfectionism variables at baseline with insomnia symptoms at follow-up.

Furthermore, baseline anxiety was significantly associated with concern over mistakes (r = .43, *p* = .01), doubts about action (r = .47, *p* = .01), parental criticism (r = .43, *p* = .01), and socially prescribed perfectionism (r = .30, *p* = .03) at follow-up. Equivalent findings were demonstrated between baseline depression with concern over mistakes (r = .35, *p* = .01), doubts about action (r = .33, *p* = .01), and parental criticism (r = .28, *p* = .03).

Moreover, concern over mistakes (r = .30, *p* = .03), doubts about action (r = .47, *p* = .01), self oriented (r = .32, *p* = .02) and socially prescribed perfectionism (r = .32, *p* = .02) at baseline were significantly associated with anxiety at follow-up. Additionally, concern over mistakes (r = .30, *p* = .03), doubts about action (r = .47, *p* = .01), personal standards (r = .47, *p* = .01) and self oriented perfectionism at baseline was associated with depression at follow-up. Finally, baseline insomnia symptoms were significantly associated with anxiety (r = .49, *p* = .01) and depression (r = .44, *p* = .01) at follow-up, similarly insomnia symptoms at follow-up was associated with anxiety (r = .56, *p* = .01) and depression (r = .42, *p* = .01) at baseline.

### Longitudinal Regression

Insomnia symptoms at baseline were positively associated with future doubts about action (step 3: 10% variance, see Table 6) after controlling for age and sex. However, this relationship was mediated by concurrent symptoms of insomnia at follow-up (step 4: 10% variance). Furthermore, anxiety remained the only significant predictor of future doubts about action in the subsequent steps (step 5: 24%; step 6: 24% variance). These results suggest that the severity of insomnia symptoms at baseline predicts an increase in future doubts about action; however, the likelihood of developing future doubts about action was mediated by previous symptoms of anxiety and current symptoms of insomnia. Of note, the pattern of results remained the same after controlling for anxiety and depression at follow-up.

**Table 6:** Longitudinal linear regression analyses with follow-up perfectionism as the dependent variables, and age, sex, baseline and follow-up insomnia severity, baseline anxiety and baseline depression as predictors.

	Dependant Variable							
	Doubts About Action				Parental Criticism			
*Predictors in the model*	R^2^	β	*t*	Sig.	R^2^	β	*t*	Sig.
Step 1								
Age	.00	-.06	-0.45	.66	.01	.04	0.28	.78
Step 2								
Age	.01	-.07	-0.46	.65	.02	.03	0.21	.84
Sex		.03	0.23	.82		.13	0.93	.36
Step 3								
Age	.10	-.07	-0.50	.61	.11	.03	0.19	.85
Sex		-.01	-0.12	.92		.08	0.62	.54
ISI Baseline		.31	2.32	.02*		.31	2.36	.02*
Step 4								
Age	.10	-.07	-0.49	.63	.17	.02	0.13	.90
Sex		-.02	-0.15	.88		.13	1.03	.31
ISI Baseline		.28	1.65	.11		.51	3.20	.01**
ISI Follow-up		.05	0.26	.80		-.34	-2.04	.05*
Step 5								
Age	.24	-.07	-0.55	.59	.30	.02	0.18	.86
Sex		-.02	-0.18	.86		.13	1.04	.30
ISI Baseline		.15	0.90	.37		.42	2.66	.01**
ISI Follow-up		-.14	-0.79	.43		-.50	-3.03	.01**
Anxiety		.47	3.10	.01**		.41	2.84	.01**
Step 6								
Age	.24	-.07	-0.54	.59	.30	.01	0.12	.91
Sex		-.02	-0.18	.86		.14	1.11	.27
ISI Baseline		.15	0.86	.39		.40	2.57	.01**
ISI Follow-up		-.14	-0.79	.44		-.52	-3.08	.01**
Anxiety		.47	2.62	.01**		.37	2.23	.03*
Depression		.00	-0.03	.97		.10	0.67	.53

Notes

ISI, Insomnia Severity Index

*Significant for p-level < .05.

** Significant for p-level < .01.

Further, insomnia symptoms at baseline were positively associated with future parental criticism (step 3: 11% variance, see Table 6) after controlling for age and sex. Interestingly this relationship was not mediated by symptoms of insomnia at follow-up, suggesting that severity of insomnia symptoms at baseline directly predicts future parental criticism (step 4: 17% variance). After controlling for baseline anxiety and depression in the subsequent steps, baseline anxiety and insomnia symptoms at both time points remained significant predictors of future parental criticism, and both together accounted for the majority of variance (step 5: 30%; step 6: 30% variance). These results suggest that previous symptoms of insomnia directly predict future parental criticism, and that this relationship is not solely mediated by previous symptoms of anxiety. Of note, the pattern of results remained the same after controlling for anxiety and depression at follow-up.

## Discussion

The present study investigated the extent to which associations between perfectionism and insomnia symptoms were mediated by anxiety and depression, concurrently and longitudinally. The current results provide further evidence that different facets of perfectionism, previously not investigated in the current context, are associated with insomnia symptoms within a general population sample. Specifically, greater doubts about action (e.g., “I usually have doubts about the simple everyday things that I do”) and parental criticism (e.g., “My parents never tried to understand my mistakes”) were associated with higher insomnia symptoms at baseline. Furthermore, these associations were at least partially mediated by anxiety and depression. Despite this, there were no associations between dimensions of Hewitt & Flett’s conceptualization of perfectionism and insomnia symptoms at neither time point. Although previous symptoms of perfectionism were not related to increases in insomnia symptoms at follow-up, the present findings indicated that the severity of insomnia symptoms at baseline was related to an increase in the reporting of the perfectionism dimensions doubts about action and parental criticism at follow-up. Additionally, these relationships appear to be mediated by symptoms of anxiety, but not depression.

The concurrent relationship between the perfectionism dimension of doubts about action and insomnia appears to be consistent throughout the literature to date [13, 16]. Various explanations have been offered for this relationship. Vincent and colleagues [13] note that those who excessively doubt their own actions may subsequently experience an elevated level of arousal during the pre-sleep period, which is a known factor in relation to delayed sleep onset [33]. Alternatively, when faced with poor sleep, it may be posited that individuals who are high in such facets of perfectionism consequently spend a disproportionate amount of time critically evaluating their sleep, leading to an elevated pre-sleep arousal.

Interestingly, the results indicate that the relationship between baseline insomnia and future doubts about action appears to be mediated by preceding symptoms of anxiety alongside current symptoms of insomnia. These conclusions support existing evidence (i.e. [30]) that the relationship between disturbed sleep and perfectionism is partially mediated by symptoms of anxiety. That said, this study differs in that it was insomnia symptoms that predicted higher perfectionistic thinking as opposed to the converse. In this context, it is possible that disturbed sleep exacerbates a preoccupation with the negative consequences and daytime impairment associated with a poor nights sleep, consequently perpetuating an overly critical self-evaluation in the perspective of sleep (i.e. an accentuation of perfectionistic tendencies). The conscious awareness that a poor nights sleep would hinder daytime functioning may then increase the likelihood of doubts over actions (i.e. “I usually have doubts about the simple everyday things that I do”). It could also be proposed that symptoms of anxiety increase the likelihood that individuals exhibit such negative doubts regarding past and future behaviour(s). Thus, these results could suggest that treatments for insomnia should address symptoms of anxiety with the prospect of reducing perfectionistic thinking, specifically doubts about action.

However, it is important to note that several of the items from the F-MPS subscale of doubts about action originate from an existing measure of obsessionality [34]. Indeed, doubts about one’s action may inherently be reflective of obsessional behaviour(s) and thinking, as well as a form of anxiety in itself. As such, this overlap may potentially account for the relationship between doubts about action and symptoms of anxiety, which appear to be exacerbated by poor sleep.

The present findings also suggest that future parental criticism appears to be predicted by symptoms of insomnia at baseline and this relationship is partially mediated by symptoms of anxiety. Although previous research has indeed noted associations between parental criticism and insomnia, the current findings, from a longitudinal aspect, shed light in respect to how symptoms consistent with insomnia may alter one’s perception of others [13]. Whilst it is likely that an overly critical self-evaluation is a consequence of a continuing sleep disturbance it appears to extend to perceived critical evaluation from others, at least in terms of parents. Despite this, symptoms of insomnia were not related to a future increase in socially prescribed perfectionism (i.e. “others expect highly of me”). Therefore, it is likely that an increase in perceived critical evaluation from others resultant from persistent sleep difficulties, at least in the current sample, is limited to that of one’s parents. It is relevant to note that the present sample consisted predominantly of young students who, to an extent, may be reliant upon parental support. As such, it may be posited that this may have influenced the current findings pertaining to an accentuation of perceived critical evaluation from parents due to a sleep disturbance.

The present study found no significant associations between the severity of insomnia symptoms and concern over mistakes. Despite this, evidence has highlighted significant relationships between concern over mistakes with pre-existing and the onset of insomnia in the future [30]. Furthermore individuals with insomnia have reported a greater concern over mistakes relative to healthy controls [13]. Additionally, the present study demonstrated no significant associations between symptoms of insomnia with the dimensions of self oriented, other oriented or socially prescribed perfectionism under Hewitt and Flett’s conceptualization of perfectionism. Thus, concern over mistakes and socially prescribed perfectionism do not appear to be related to reports of insomnia within the present sample.

That said, Azevedo and colleagues [14, 15] highlight noteworthy relationships between the perfectionism dimension of socially prescribed perfectionism with difficulty initiating and maintaining sleep amongst an undergraduate student sample. Vincent and Walker [13] also note a trend for individuals with chronic insomnia to report greater socially prescribed perfectionism relative to a sample of healthy controls recruited from a community sample. Thus, the current findings, or lack thereof, regarding the relationship between concern over mistakes and socially prescribed perfectionism with insomnia symptoms may be due to the examination of symptom severity across a continuous spectrum as opposed to discrete groups. As such, the perfectionism dimensions of concern over mistakes and socially prescribed perfectionism could potentially be more profound amongst individuals with clinically diagnosed insomnia.

The present study used a comprehensive assessment to address insomnia symptoms amongst the general population from the perspective of diagnostic criteria, covering not only sleep initiation or maintenance difficulty as a definition commonly used in the perfectionism/insomnia literature, but additionally examining early morning awakenings, dissatisfaction with sleep, and daytime impairment(s). Despite this, our findings should be considered as preliminary due to the limited number of respondents at baseline, and high rate of attrition at follow-up. Although significant associations were indeed established at baseline, the rate of attrition may have influenced the lack of associations at follow-up. That said, there were no differences between those who completed the survey at both time points and those who completed only the baseline assessment on ISI scores, HADS scores or any of the dimensions of perfectionism. Furthermore, although three time points are typically used in order to assess mediation in the context of a longitudinal design, this avenue was not perused in the current study due to the high level of attrition at follow-up. In addition, it is worth noting that many studies, which have examined the relationship between perfectionism and insomnia from a longitudinal approach, have also only used two time points [15, 30].

Regardless of this caveat, the present study is, to the best of the authors’ knowledge, the first to incorporate all dimensions and subscales from both conceptualizations of perfectionism in order to investigate the mediating role of anxiety and depression on the relationship between insomnia symptoms and aspects of perfectionism. Although the current findings cannot be generalized to clinically defined insomnia, the use of a general population sample can be regarded as a practical step towards the identification of the mediating role of anxiety and depression in the development of insomnia. Symptoms of, and primary mechanisms underlying, insomnia can be viewed to exist along a continuum [35]. The same processes are likely present amongst both general population and clinical samples, however divergent in terms of severity. Thus, it is possible that the pattern of associations may be conservative in a general population sample, and associations between these variables may be stronger in a sample meeting diagnostic criteria for insomnia. Accordingly, future research should examine the present research questions amongst a larger, clinical sample. Such research may add further support these preliminary results, which suggest that the relationships between symptoms of insomnia with doubts about action and parental criticism are indeed mediated by anxiety. Additionally, such research would further highlight the significance of treating symptoms of anxiety in order to relieve negative thoughts concerning doubts about action, and parental criticism with the prospect of reducing insomnia symptoms.

Furthermore, there appears to be evidence that psychological interventions, such as cognitive behavioural therapy, specifically targeting perfectionism have been associated with a decrease in symptoms of anxiety (see [36] for a review). Likewise, cognitive behaviour therapy treatments for anxiety have also been associated with a significant reduction in perfectionism [37]. As such, it would be beneficial for future research to also compare the effects of psychological interventions that differentially target anxiety and perfectionism in their potential ability to comparatively reduce symptoms of insomnia.
